# Primary Spinal Intradural Melanocytoma of the Thoracic Region: A Rare Case

**DOI:** 10.7759/cureus.41019

**Published:** 2023-06-27

**Authors:** Talha Abbas, Sakina Batool, Bireera Muzaffar, Zainab Ahsan, Fahad B Hamid, Abdul Hameed, Mah R Tariq

**Affiliations:** 1 Department of Neurosurgery, Sir Ganga Ram Hospital, Lahore, PAK; 2 Department of Internal Medicine, Sir Ganga Ram Hospital, Lahore, PAK; 3 Department of Internal Medicine, Combined Military Hospital (CMH) Lahore Medical College and Institute of Dentistry, Lahore, PAK; 4 Department of Medicine, Combined Military Hospital (CMH) Lahore Medical College and Institute of Dentistry, Lahore, PAK

**Keywords:** exophytic mass, intramedullary, intradural, spinal melanocytoma, primary

## Abstract

While the presence of metastatic melanocytoma in the central nervous system (CNS) is relatively common, primary spinal melanocytoma (PSM) is an extremely rare entity. Only 70 cases have been reported, and its usual position is the cervical region. We report a case of a 35-year-old male with primary spinal intramedullary melanocytoma with a dorsal exophytic component. The tumor was first opened in the periphery and was closed without being operated upon due to it being an uncommon pathology.

## Introduction

Primary central nervous system (CNS) melanocytoma is a rare entity, constituting roughly 1% of all melanocytoma cases. Primary spinal melanocytoma (PSM) is even more infrequent in its occurrence. While primary spinal melanocytomas are usually insidious in their onset, they have the propensity to present acutely with hemorrhage, pain, and neurological symptoms, such as paralysis. We report here a case of a 35-year-old male who was hospitalized after presenting with severe, radiating lower back pain, numbness of his lower limbs, and paraplegia for 20 days, as well as fecal and urinary incontinence.

## Case presentation

**Figure 1:**
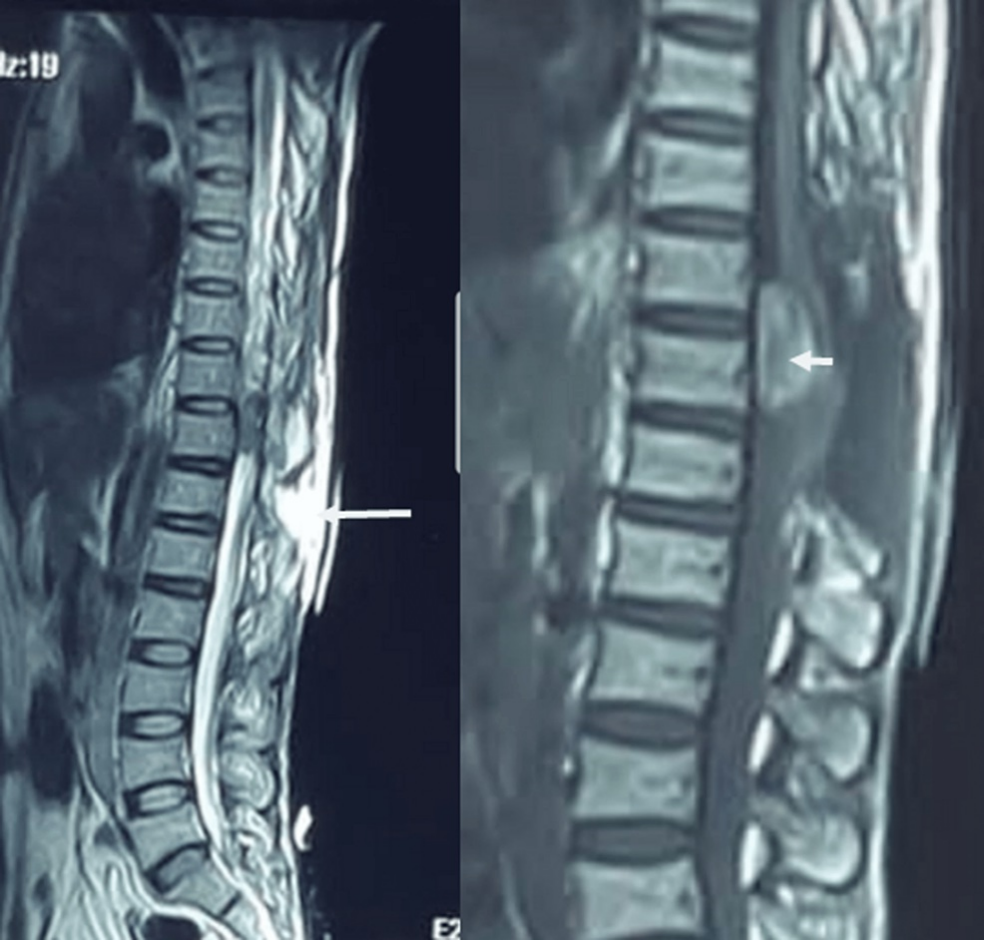
Magnetic resonance imaging image on the left shows a mass at D9/D10 levels. The image on the right shows a yellowish, exophytic mass present ventral to the cord, pushing it dorsally.

A 35-year-old male presented to the emergency department with a complaint of watery discharge from spinal surgery wound. He had sudden onset of paraplegia for 20 days, which was associated with severe lower back pain radiating to both lower limbs, as well as numbness of both lower limbs for the past 3-4 days. He was previously operated on at another hospital for a spinal space-occupying lesion two weeks before but was then referred to our department, with the aforementioned complaints as well as fecal and urinary problems. Magnetic resonance imaging (MRI) of the dorsal spine was acquired, which showed loss of posterior spinal elements most probably due to previous surgery. There was a space-occupying lesion lying adjacent to D10 and D11 vertebral bodies, ventral to the cord and pushing it backward (Figure 1). It was hyperintense on the T1-weighted image but hypointense on the T2-weighted (T2W) image. A pseudo-meningocoele was also noted on T2W MRI.

Other than the surgery mentioned, his past medical, surgical, social, and personal history was unremarkable. Physical examination of the lower extremities bilaterally revealed a power of 0/5. Plantar, ankle, and anal sphincter reflexes were absent. Dermatological and eye examinations were unremarkable, which was crucial in helping to rule out melanoma of a skin or ophthalmologic origin.

**Figure 2:**
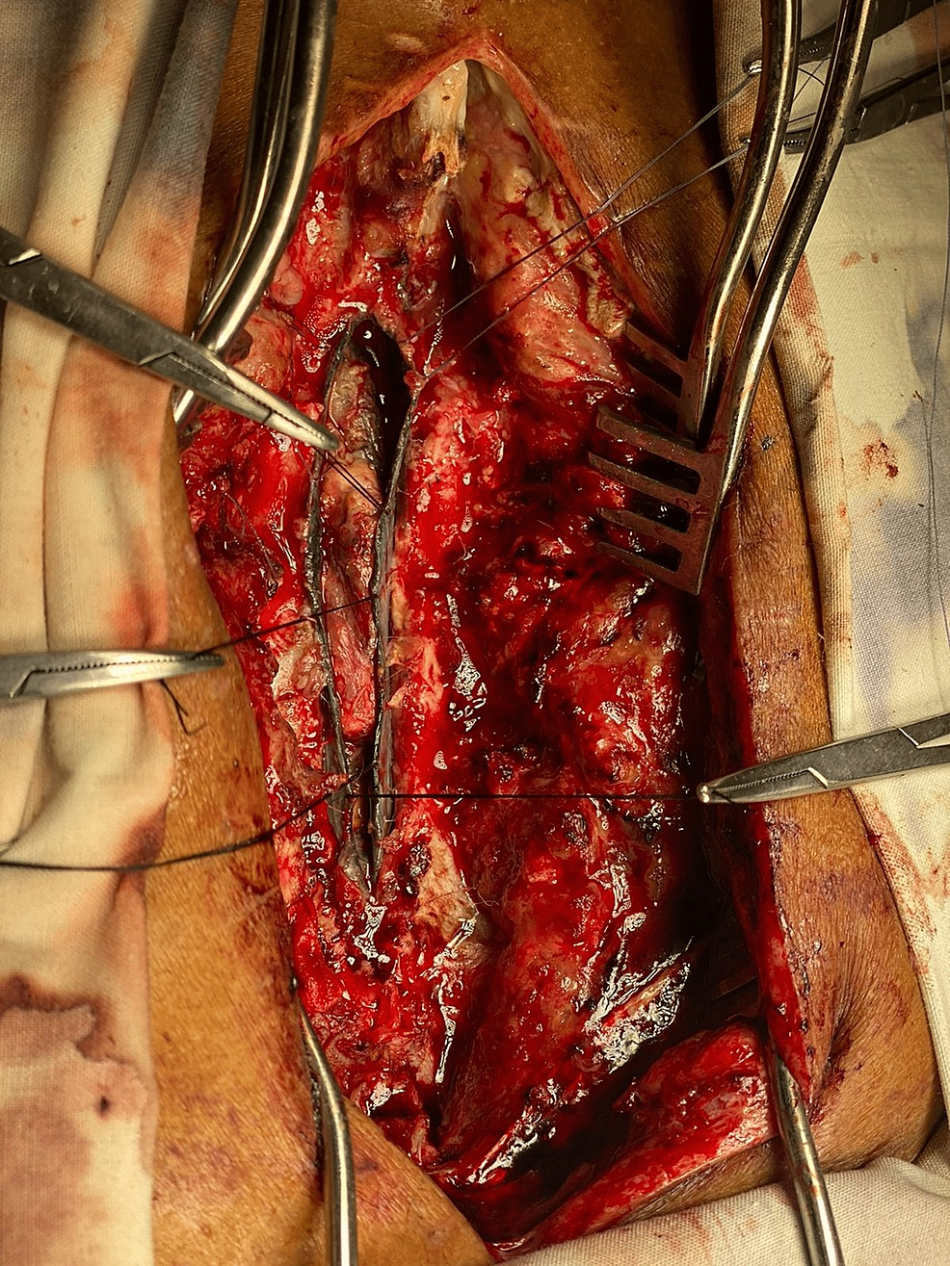
Post-laminectomy and pre-excisional findings showing an intradural, intramedullary mass, with black dura and black arachnids.

As there was a frank cerebrospinal fluid (CSF) leak, microbiological investigations were done, which were insignificant, with a negative culture report. Exploration of the previous wound was planned primarily for CSF repair and excision of the spinal space-occupying lesion. Under general anesthesia, the previous incision was opened. At first glance, unusual pathology revealed itself as large epidural venous channels; this was also written in the operation notes of the previous surgeon. After clearing fields, these venous channels were revealed to be folded ends of black-pigmented opened dura. This black pigmentation was spread over all leptomeninges (Figure 2). The dura and arachnoid were pigmented black, whereas the pia made the appearance of the cord dirty yellow. The cord at D10/D11 segments was pushed dorsally by a black-colored lesion attached to the ventral side of the cord. It was intradural but exophytic on the dorsal aspect of the cord, more on the left side. The cord at this level seemed to be a compact hardened nidus of black-colored malformed vessels of small supra-capillary size.

**Figure 3:**
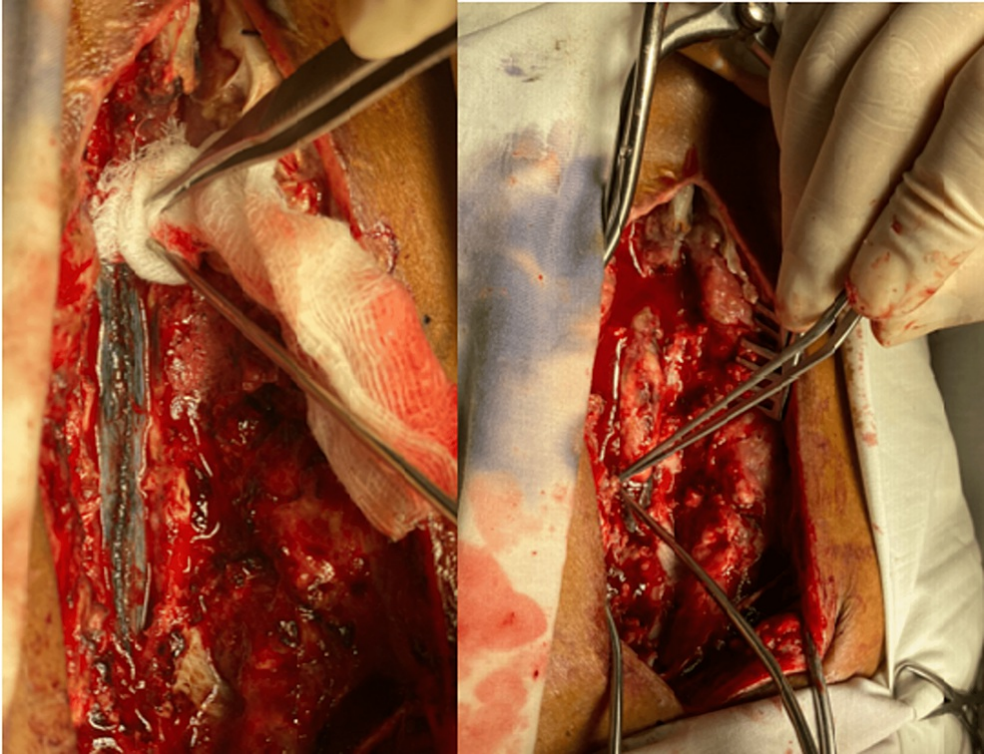
Operative procedure.

Through a trans-dentate approach, the exophytic lesion was removed by a cavitron ultrasonic surgical aspirator (CUSA) and suction (Figure 3), following which hemostasis was secured. The dura was closed by Vicryl 4/0, and fibrin glue was applied after the Valsalva maneuver and noting subsequent cord pulsations. Spongostan was placed. The wound was then thoroughly washed with hydrogen peroxide and normal saline and was closed in reverse order. Retention sutures were applied, and a drain was placed. An excision biopsy specimen was sent for histopathology.

**Figure 4:**
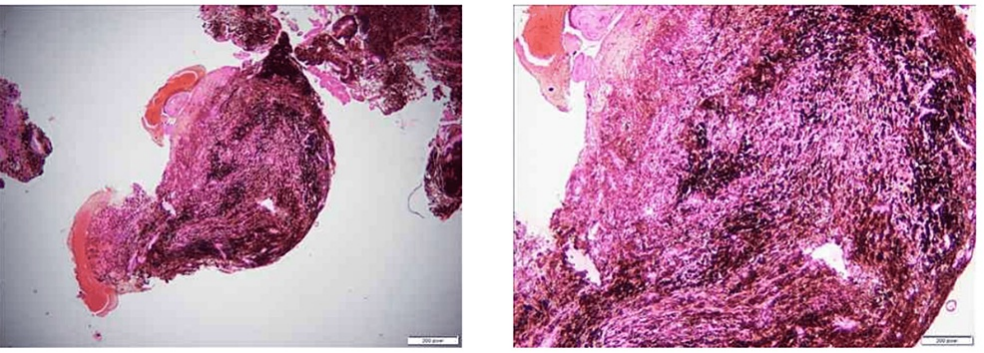
Histological examination of the section reveals multiple fragments comprising areas of hemorrhage and the lesion composed of spindle cells and dense areas of pigment deposits.

The specimen sent for histopathological examination comprised multiple blackish soft tissue fragments measuring 1 × 1 cm, which were marked by hemorrhagic areas and were all passed in one block (Figure 4). The microscopic examination revealed a cellular, heavily pigmented neoplasm composed of spindle cells and dense deposits of black pigment. Areas of necrosis and atypical mitosis were not identified. Differential diagnoses included pigmented schwannoma, pigmented meningioma, and melanocytoma.

**Figure 5:**
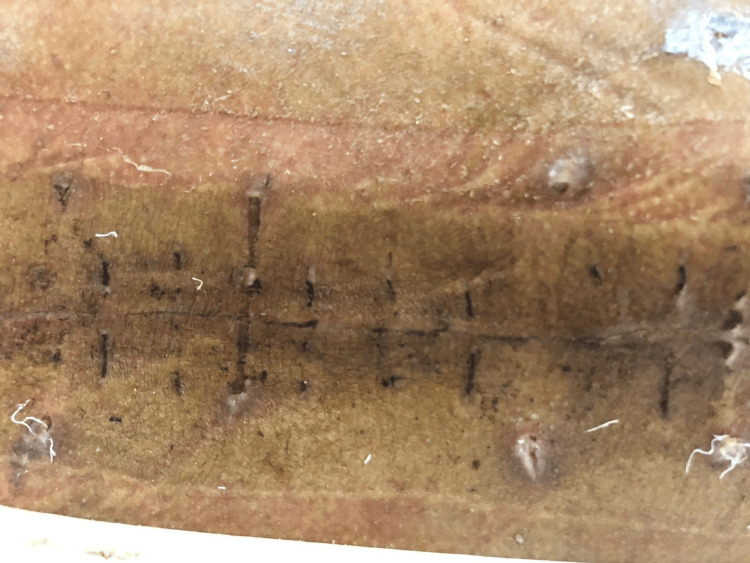
Post-surgical scar.

The sample was sent for immunohistochemical analysis, the results of which are described as follows: the tumor cells stained positive for Melan-A and S-100 and negative for epithelial membrane antigen (EMA) and progesterone receptor (PR), and the diagnosis for the spinal space-occupying lesion was given as a melanocytoma. A post-surgical scar remained (Figure 5), with appropriate scar care advised.

The patient was hospitalized for 12 days following the surgery, during which he was found to be vitally stable. Bladder wash and suprapubic cystostomy were scheduled, and no active issue was reported during the hospital stay. Power gradually increased to 1/5 and is expected to increase even more with time. Postoperative radiotherapy/chemotherapy was not carried out due to financial constraints. Appropriate vocational and psychological rehabilitation was advised as the patient appeared to be visibly distressed by the possible impact of his diagnosis on the social and work aspects of his life. Follow-up MRI after three months revealed complete excision of the exophytic component of the tumor with partial recovery of sensations in lower limbs, but no significant improvement in motor power.

## Discussion

Primary CNS melanocytomas have been found to be extremely rare, accounting for roughly 1% of all cases, with primary spinal cord melanocytomas proving to be even rarer. Intradural intramedullary tumors have been found to be infrequent even among primary spinal cord melanocytomas, making up about 37.7% of the primary spinal cord melanomas so far reported [1]. Primary melanocytoma in the CNS has two primary sources of origin: either from the melanoblasts that accompany the pial sheaths of the vascular bundles or from neuroectodermal rest cells during embryogenesis [2,3].

The clinical presentation of PSM overlaps with neurofibromas, meningiomas, and ependymomas due to its non-specificity in terms of clinical presentation [4-6]. Commonly, the patient presents with back or neck pain along with progressive, asymmetrical myelopathy [7]. Our patient slightly deviated in this presentation since he presented with bilateral lower limb weakness instead of it being asymmetric. For the purposes of diagnosis, spinal MRI is considered the gold standard owing to the fact that melanin’s paramagnetic properties or hemorrhagic elements in the tumor make it very sensitive [8]. Most reports have shown that the MRI pattern of spinal cord melanocytoma includes signal hyperintensity on T1-weighted images and signal iso- or hypointensity on T2-weighted images. Our MRI report was in line with this general finding. The MRI signal of melanocytic tumors depends on the presence of melanin, acute or chronic intra-tumoral hemorrhages, and fat deposits [9-11]. The characteristic MRI appearance due to the presence of pigment helps to distinguish PSM from other spinal tumors such as schwannoma. However, an MRI fails to separate a primary malignant melanocytoma from other pigmented lesions found in the CNS, for example, leptomeningeal melanoma and metastatic malignant melanoma [12]. In such cases, an accurate diagnosis is only possible by histopathological examination. Relevant histopathological tests including for HMB-45 and S-100 proteins give a positive result by demonstrating immunoreactivity and help in establishing a definitive diagnosis. Moreover, microscopic examination reveals the formation of tight nests surrounded by well-differentiated melanocytes with cytoplasm rich in melanin. These findings collectively help in establishing a diagnosis [13].

Hayward [2] published the most widely accepted criteria for the diagnosis of primary CNS melanocytoma: (1) no malignant melanocytoma identified outside the CNS, (2) intramedullary spinal lesions, (3) involvement of the leptomeninges, (4) single intracerebral lesion, (5) tumor in the pituitary or pineal gland, and (6) hydrocephalus. These criteria, along with other clinical features and histopathological confirmation, usually allow for the diagnosis of primary CNS melanocytoma. Our case fulfills these criteria.

Various treatment options can be suggested for PSM; however, a definitive treatment with stated protocols is currently unavailable. The treatment of choice in most cases is surgical resection as was performed in our case. The treatment approach for PSM varies greatly from metastatic melanoma, hence the need for a correct diagnosis. Surgical resection not only removes the tumor but also helps in establishing the final diagnosis. In addition to the surgical approach, adjuvant radiotherapy may help in limiting tumor dissemination [14].

## Conclusions

Primary spinal melanocytoma is an extremely rare disease, and diagnosing it includes eliminating all other possible causes of the mass and moving toward appropriate treatment and rehabilitation. Owing to the rarity of the condition, it is imperative to create greater awareness about its existence. This will enable a more prompt diagnosis and lead to minimal undesirable sequelae.
